# Building capacity in dissemination and implementation science: a systematic review of the academic literature on teaching and training initiatives

**DOI:** 10.1186/s13012-020-01051-6

**Published:** 2020-10-30

**Authors:** Rachel Davis, Danielle D’Lima

**Affiliations:** 1 Centre for Implementation Science, Health Service and Population Research Department, King’s College London, London, UK; 2 Centre for Behaviour Change, Department of Clinical, Educational and Health Psychology, University College London, London, UK

**Keywords:** Dissemination and implementation science, Capacity building, Training opportunities, Knowledge translation

## Abstract

**Background:**

The field of dissemination and implementation (D&I) science has grown significantly over recent years. Alongside this, an increased demand for training in D&I from researchers and implementers has been seen. Research describing and evaluating D&I training opportunities, referred to here as ‘capacity building initiatives’ (CBIs), can help provide an understanding of different methods of training as well as training successes and challenges. However, to gain a more detailed understanding of the evidence-base and how D&I CBIs are being reported in publications, a field-wide examination of the academic literature is required.

**Methods:**

Systematic review to identify the type and range of D&I CBIs discussed and/or appraised in the academic literature. EMBASE, Medline and PsycINFO were searched between January 2006 and November 2019. Articles were included if they reported on a D&I CBI that was developed by the authors (of each of the included articles) or the author’s host institution. Two reviewers independently screened the articles and extracted data using a standardised form.

**Results:**

Thirty-one articles (from a total of 4181) were included. From these, 41 distinct D&I CBIs were identified which focussed on different contexts and professions, from 8 countries across the world. CBIs ranged from short courses to training institutes to being part of academic programmes. Nearly half were delivered face-face with the remainder delivered remotely or using a blended format. CBIs often stipulated specific eligibility criteria, strict application processes and/or were oversubscribed. Variabilities in the way in which the D&I CBIs were reported and/or evaluated were evident.

**Conclusions:**

Increasing the number of training opportunities, as well as broadening their reach (to a wider range of learners), would help address the recognised deficit in D&I training. Standardisation in the reporting of D&I CBIs would enable the D&I community to better understand the findings across different contexts and scientific professions so that training gaps can be identified and overcome. More detailed examination of publications on D&I CBIs as well as the wider literature on capacity building would be of significant merit to the field.

**Supplementary information:**

**Supplementary information** accompanies this paper at 10.1186/s13012-020-01051-6.

Contributions to the literature

- Identifying training opportunities in dissemination and implementation (D&I) in the published literature can help shed light on training successes, challenges and gaps.
- We provide a field-wide perspective on the type and range of D&I training opportunities and how these are reported on through systematically reviewing the academic literature
- Training priorities are identified alongside challenges of building capacity in the field. These are of use to consider in efforts to develop future D&I training endeavours.

## Introduction

The failure to optimally use research to improve population outcomes and reduce service inefficiencies is an endemic challenge to health and social care systems worldwide [1–3]. A critical and acknowledged issue is the considerable gap between what we know we should be doing based on the evidence, versus what gets implemented in healthcare settings [3, 4]. Dissemination and implementation science (referred to hereon in, as ‘D&I’) investigates ways to close ‘research to practice’ gaps (*‘implementation science’*) and spread knowledge and information to practice settings (*‘dissemination science’*) [5, 6].

The critical role of D&I in enhancing the application of evidence-based interventions has led to the discipline’s rapid advancement in recent years [7, 8]. Significant steps have been taken to build D&I capacity (defined as ‘*a process which leads to greater individual, organisation or system capabilities to conduct and implement high-quality research and* practice’ [9–12]) in recognition that a robust and sustainable workforce is required to successfully implement or maintain health and social care interventions of known effectiveness [13, 14].

Efforts to build capacity in D&I take many forms [15–18]. In the USA, as early as 1998, research organisations and initiatives were established (e.g. the Veterans Health Administration *‘Quality Enhancement Research Initiative’* (QUERI) [19–21]), with the aim of investigating ways to efficiently implement research-driven best practices. Academic and government institutions, centres and departments dedicated to the field have since been created in the USA [22–26], Canada [27, 28], Australia [29, 30], the UK [31, 32] and other countries [33–35] as well as global efforts [36]. Opportunities for D&I funding are increasingly available [7, 37–39], professional societies and groups have been set up [40–42] and there are a growing number of scientific conferences and meetings [43–47]. In 2006, the specialist journal ‘*Implementation Science*’ was born and, in 2019, the inception of its companion journal (*Implementation Science & Communications*) as well as several other journals and libraries that have developed over the years (e.g. the Cochrane library) that publish D&I-related research [7].

Alongside these efforts, another very important way to build D&I capacity is through the development and delivery of teaching and training initiatives—referred to hereon in as ‘*capacity building initiatives’* (CBIs). These endeavours may be aimed at individuals conducting research (i.e. *‘researchers’*), those faced with translating evidence into practice (i.e. *‘implementers’*) [15–18] or those tasked with training others in D&I principles and methodologies (i.e. *‘educators’*). Such training endeavours include short courses, workshops and webinars or they may form part of academic programmes [16–18, 46–49]—all of these can be important in ensuring individuals have the requisite knowledge and skill-set to successfully implement scientific discoveries across diverse populations [14–17, 50, 51]. Given the value of D&I CBIs, it is of interest to examine the type and range of training opportunities available [15–18] and how these extend to a wide range of individuals (implementers, researchers and educators) [14, 17, 52–56].

In 2015, *Implementation Science* expressed a renewed interest in research describing and critically appraising D&I training initiatives [52]. Since this editorial, several descriptive and/or evaluative articles on D&I CBIs have been published [18, 47, 49, 57, 58], but for those working in D&I to gain a more detailed understanding of the evidence-base, a field-wide perspective of the published literature is required [59]. A useful starting point to address this gap is through the review and documentation of D&I CBIs that have been written up in the academic literature. Examining the way in which training endeavours are reported can help highlight variabilities in reporting and enable comparisons of different CBIs against set criteria (e.g. mode of delivery, duration, target audience) so that gaps in training (and the reporting of training) can be identified.

To date, several articles published between 2013 and 2019 have reviewed (at least in part) D&I CBIs and resources, specifically related to teaching and training. In 2013, an article that focussed on developing the next generation of implementation researchers highlighted selected D&I training programmes, conferences and resources [55]. In 2017, a mapping exercise of D&I research training initiatives, stemming from the National Institute of Health’s 2013 meeting on training, measurement and reporting was published—comprising training institutes, academic programmes and courses, webinars and career development awards [17]. In the same year, an expert assessment on training opportunities in D&I was documented [59] together with a content analysis of D&I resources using public, web-based information [60]. More recently (in 2018–2019), studies have identified D&I training initiatives to help inform medical education [61], training needs in public health [62] and mental health [16], and a review of online D&I resources was performed [63]. Taking this evidence collectively, the value of D&I CBIs in developing and harnessing skills in implementation research and evidence translation can be seen. Taking the evidence individually, however, most of the research is geographically restrictive, focussing only on D&I CBIs in the USA [59] or the USA and/or Canada [16, 55, 61, 63]. While one paper considered D&I training efforts on a global level [62], this was not the main aim of the work, and thus, information on the characteristics of the CBIs and gaps in training needs were understandably limited.

With these thoughts in mind and heeding the call from *Implementation Science* on the need for publications on D&I CBIs [52], we present the findings of a systematic review aimed at identifying the type and range of D&I training opportunities reported in the academic literature. This review is part of a larger programme of work aimed at describing and appraising D&I CBIs. The aim of this paper is to provide a detailed description of our review methodology and a high-level summary of the main features and characteristics of the training initiatives and how these are reported. We also reflect on the implications of our findings and put forward recommendations on the future reporting of CBIs in the context of D&I science.

## Methods

### Search strategy

EMBASE, MEDLINE and PsycINFO were searched (using the OVID interface) for relevant articles published between January 2006 and November 2019. The cut-off point was set at 2006 in line with the inception of *implementation science* [64]—where most of the relevant articles identified in our initial scoping of the literature were published. The search strategy was informed by several reviews and discussion papers on D&I-related terms [65–69] together with a brainstorming exercise involving both authors (RD, DD) to generate applicable terminology. Terms relating to (1) implementation science (e.g. ‘knowledge translation’, ‘implementation research’) and (2) teaching and training (e.g. ‘capacity building’, ‘curriculum’) were included. To avoid a priori assumptions on the type of content (i.e. topics) the CBIs may cover, the search strategy was restricted to generic terms relating to D&I (e.g. ‘implementation science’) rather than specific terms that focussed on D&I methodologies or concepts (e.g. ‘hybrid designs’ [70], ‘implementation outcomes’ [71], or theories and frameworks, e.g. ‘Consolidated Framework for Implementation Research’ [72]). To tighten the search specificity, the search strategy was customised using appropriate wildcards (e.g. course$) and Boolean operators (i.e. OR, AND), and restricted to titles and abstracts. The sensitivity of the search was assessed by forward and backward citation searching of included articles and through handsearching key implementation and behavioural science journals (e.g. *Implementation Science, Translational Behavioural Medicine*). The final search was conducted on 21st November 2019 (see Table 1 for a full list of search terms).

**Table 1:** Search strategy

Search facets	Terms
Facet 1: Terms relating to implementation science	(dissemination science OR implementation research OR implementation science OR improvement research OR improvement science OR knowledge mobilisation OR knowledge transfer OR knowledge translation OR quality improvement).ti,ab.
	Limit to English Language
	Limit to humans
	Limit to 2006-current
Facet 2: Terms relating to teaching and training	(capacity building OR course OR competencies OR curriculum OR lecture OR seminar OR teach OR training OR webinar OR workshop).ti,ab.
	Limit to English language
	Limit to humans
	Limit to 2006-current
1 AND 2	Remove duplicates

### Inclusion criteria

At the first stage of screening (title and abstract), any empirical or review article that discussed CBIs in D&I and/or related areas (e.g. ‘improvement science’, ‘quality improvement’, ‘translational research’) in the context of teaching or training was included. At the second stage of screening (full text), tighter restrictions applied. Articles whereby authors discussed or appraised (as a primary or secondary focus) a D&I CBI they (or their host institution) developed were included—this comprised CBIs where the whole focus of the training was on D&I (e.g. a D&I workshop) or only part of the focus (e.g. a D&I module that formed part of a larger postgraduate programme in public health). Articles were not restricted based on their methodological focus—in other words, we included all D&I CBIs that met our inclusion criteria, irrespective of the type of information provided on the D&I CBI or the level of detail.

### Exclusion criteria

Dissertations and doctoral theses, books/book reviews, conference posters/presentations and editorials/commentaries were excluded, as were articles not in English. Review papers were excluded following citation searching for relevant articles, as were articles that focussed on training in other areas of healthcare improvement (e.g. patient safety or quality improvement) if they did not include an element of D&I within the training (e.g. [73, 74]). Articles were also excluded if they: described D&I-related conferences or conference proceedings (e.g. [56, 75, 76]) unless there was a specific D&I CBI within the conference (e.g. workshop) that delegates could register for, examined how D&I methodologies or knowledge translation techniques could be used to better implement training programmes [77] or training centres [78] without the focus of the training itself being on D&I and assessed D&I training needs [14, 55, 79] or competencies in D&I [80] or discussed the development of D&I-related research centres [21] without reference to a specific D&I CBI. Equally, articles that provided an overview of a meeting(s) to discuss how to advance the field of D&I [81, 82], focussed on the development of collaboratives to encourage new research partnerships [83], presented general repositories for D&I resources or training opportunities [60, 63] or calls from journals for work relating to D&I CBIs [23] were excluded.

Finally, articles that focussed on the development of training programmes for mentors working in translational science [84, 85] were excluded unless the content of the mentoring was on D&I science (versus more generally on how to be an effective mentor), as were those that explored mentoring approaches as a way of assisting knowledge translation without actually discussing a D&I-related mentoring scheme [86].

### Screening of articles

Articles were screened for relevance by the lead author (RD). The second author (DD) independently screened a random selection of 20% of the articles at the first stage of screening (title and abstract) and 100% at the second stage (full text). Discrepancies were resolved through discussions between the authors until consensus was reached.

### Data extraction

A standardised form was developed to extract data from the included articles and to help synthesise the data in the review. The ‘Template for Intervention Description and Replication’ (TIDieR) checklist [87] was used as a starting point to see which items in the checklist would be of relevance to review aims—the TIDieR specifies the clear reporting of interventions (in our context ‘training interventions’). Additional criteria of potential relevance were identified by the lead author (RD) and agreed by the second author (DD) by searching google scholar, electronic databases (e.g. PUBMED) and consulting with the Equator network website for relevant guidelines (https://www.equator-network.org/). Operational definitions for each criterion were developed and tested across all included articles to ensure reliability, validity and consistency in the data extraction process (see Suppl. file 1 for data extraction form).

For articles where authors discussed more than one D&I CBI they had developed (i.e. they presented a suite of D&I CBIs that were independent of one another, such as workshops or postgraduate courses), data was extracted on each CBI separately. Data was extracted by both authors (RD, DD) across all articles to ensure consistency and accuracy in the reporting of findings.

### Quality assessment

The eligibility criteria for articles in our review led to the inclusion of heterogenous research in terms of aims and methodological focus. It was deemed inappropriate therefore to appraise the methodological quality of the articles. Instead, we used the data extraction form (Suppl. file 1) to describe key characteristics of the D&I CBIs, delineate commonalities and differences between these and highlight key learnings when taking the evidence collectively.

## Results

### Search results

**Fig. 1:**
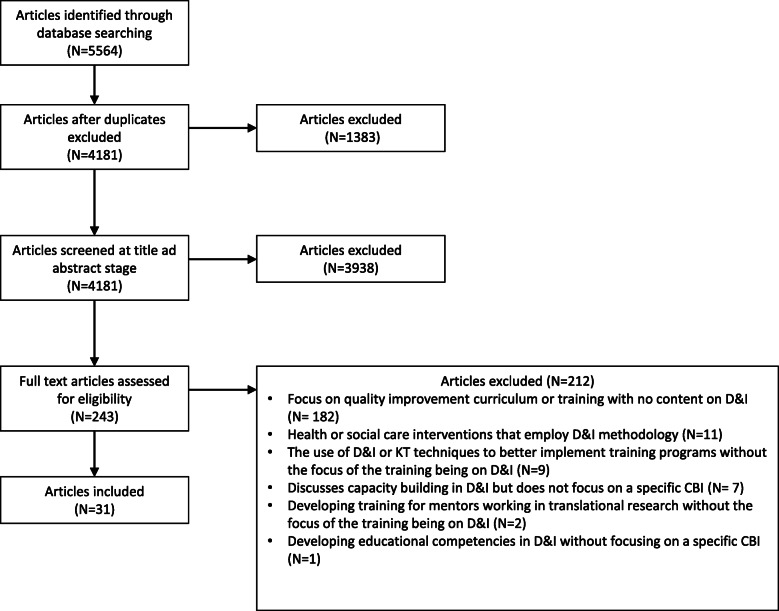
PRISMA flowchart of results

The search retrieved 5564 articles, with a total of 4181 remaining after the removal of duplicates (*N* = 1383). A further 3938 articles were excluded at the title and abstract stage, resulting in 243 full-text articles assessed for eligibility. Of these, 212/243 articles were disregarded (see Fig. 1 for reasons), leaving 31 articles relevant for inclusion.

There was a high level of agreement (> 90%) regarding inclusion between the reviewers (RD and DD) at both stages of screening, with disagreements quickly and easily resolved.

#### Key characteristics of included articles

Articles spanned a 13-year period (2006–2019) with the majority published during or post 2014 (*N* = 21) [18, 47, 57, 58, 62, 88–103] and 10 articles published pre 2014 (*N* = 10) [48, 49, 104–111]. Publications originated from 8 countries: the USA (*N* = 21) [18, 48, 57, 58, 62, 88–90, 92–96, 98–101, 103, 104, 108, 109], Canada (*N* = 3) [106, 107, 110], Australia (*N* = 2) [49, 91], the UK (*N* = 1) [105], Sweden (*N* = 1) [47], Kenya (*N* = 1) [97], Germany (*N* = 1) [102] and Saudi Arabi (*N* = 1) [111].

#### Articles reporting on the same CBI

Most of the articles (*N* = 27) [47–49, 57, 58, 62, 88, 89, 91–103, 105–109, 111] reported on just one CBI, with the remaining articles (*N* = 4) [18, 90, 104, 110] discussing between 2 and 10 different D&I CBIs. Four CBIs were the focus of more than one article, including the Implementation Research Institute (IRI) (mentioned in 4 articles) [18^a^, 57, 92, 109], the Knowledge Translation Summer Institute (KTSI) (mentioned in 3 articles) [106, 107, 110^c^], the Mentored Training for Dissemination and Implementation Research in Cancer (MT-DIRC) (mentioned in 2 articles) [18^b^, 98] and the Training in Dissemination and Implementation Research in Health (TIDIRH) (mentioned in 2 articles) [48, 103]. Considering the articles separately, 48 CBIs were reported, but taking the articles collectively, accounting for those CBIs that were reported in more than one article, 41 distinct CBIs were reported (across the 31 included articles).

#### Key characteristics of the included D&I CBIs

For the remainder of the results, findings are presented in relation to the number of included D&I CBIs (*N* = 41). Where CBIs are discussed in multiple articles (e.g. data is drawn from two articles on the TIDIRH) [48, 103], this is reflected in the number of references accompanying each finding.

Of the 41 D&I CBIs identified, a range of ‘types’ of training (as defined by the authors of each of the included articles) were reported including training Institutes (*N* = 4) [18^a^, 48, 57, 92, 103, 106, 107, 109, 110^c^] or courses that were part of training programmes (*N* = 2) [18^b^, 98, 100] or training initiatives (*N* = 1) [99]; workshops (*N* = 4) [49, 93, 95, 111], seminars (*N* = 3) [90^a^, 110^a,b^], clerkships (*N* = 1) [88], mentorship programmes (*N* = 1) [90^b^], graduate certificates (*N* = 1) [89], webinars (*N* = 1) [90^c^], fellowship programmes (*N* = 1) [97], master’s programmes (*N* = 1) [102] or modules that have been integrated in master’s programmes—including clinical research (*N* = 1) [104^a^] primary health care (*N* = 1) [105] and public health (*N* = 1) [62]; or PhD programmes (*N* = 1) [94] or modules that are integrated as part of a Doctors of Nursing programme (*N* = 2) [101, 108]. The 15 remaining CBIs were termed by the authors as ‘courses’ relating (in part or in full) to D&I science [18^c,d^, 47, 58, 91, 96, 104^b,c^, 110^d-k^].

Fourteen CBIs were delivered face-face [47–49, 88, 91, 95, 100–104^a^, 106, 107, 110^a-c,i,k^, 111], 7 were delivered remotely (either online, over the phone or through video-conferencing) [62, 90^a-c^, 105, 110^a,b^], 8 were blended—employing F-F and remoted-based methods [18^a,b^, 57, 58, 89, 92, 96–98, 108, 109], 4 were delivered either F-F or remotely (i.e. individuals picked one mode of delivery) [93, 110^e,f,h^] and for the remainder, it was not reported/not clear (*N* = 8) [18^c,d^, 94, 104^b,c^, 110^d,g,j^]. CBIs ranged in length from hours [93], day(s) [49, 91, 106, 107, 110^g^], week(s) [11, 48, 103], month(s) [47, 58, 88, 96, 108] to years [18^a,b^, 57, 89, 92, 97–99, 102, 109].

Tables 2 and 3 provide further information on some of the key selected characteristics of the included CBIs.

**Table 2:** Characteristics of the D&I CBIs included in the review

First author/date	Lead organisation	Name of D&I CBI	What^a^/how	Description	Level
Ackerman, 2016 [88]	University of California, USA	1. Action Research Programme (ARP)	Clerkship/F-F	Nine-month CBI comprising experiential learning in cardiology clinics (weekly in sequential rotations of 9 consecutive weeks), reflective writing (after each clinic rotation), seminars on systems-based practice and implementation science (weekly in the first 3 months of training), and a clinic-based project focussed on strategies to improve the quality and efficiency of clinical operations (amount of time dedicated to this at the students’ discretion). CBI includes a mentoring component from clinicians.	UG
Baumann, 2019 [57] (e-print ahead of 2020 publication)	Washington University, USA	2. Implementation Research Institute (IRI)	Training institute/**blended**	Two-year CBI comprising 2 annual week-long training sessions in mental health implementation science, plus mentoring throughout the course of the training. **Also involves working on a concept paper, field implementation projects and attending implementation science conferences.** This paper focusses on the productivity of those that have attended the training. **CBI also described in Brownson, 2017A, Luke, 2016, Proctor, 2013.**	**Doct**
Brownson, 2017A [18]	Washington University, USA	2. Implementation Research Institute (IRI)	Training institute/**blended**	Two-year CBI comprising 2 annual week-long training sessions in mental health implementation science, plus mentoring throughout the course of the training, **attending implementation science conferences, working on a concept paper and field implementation projects. CBI also described in Baumann, 2019, Luke, 2016 and Proctor, 2013.**	**Doct**
Brownson, 2017B [18]	Washington University, USA	3. Mentored Training for Dissemination & Implementation Research in Cancer (MT-DIRC)	Training programme/**blended**	Two-year CBI comprising 2 annual 5 day-long training sessions and mentoring over the full duration of the training, **working on a research proposal or project and webinar sessions. CBI also described in Padek, 2018.**	**PD**
Brownson, 2017C [18]	Washington University, USA	4. Introduction to D&I Science	Course/NR	One credit course—highlighted in the article in the ‘Dissemination and Implementation Research Core’ section but no further information provided.	NR
Brownson, 2017D [18]	Washington University, USA	5. Implementing and Evaluating Evidence-based practice	Course/NR	Three credit course—highlighted in the article in the ‘Dissemination and Implementation Research Core’ section but no further information provided.	NR
Burton, 2016 [89]	University of Florida, USA	6. The Institute for Translational Research in Adolescent Behavioural Health	Graduate certificate/blended	15 credit graduate certificate which is held over 4 consecutive academic semesters and comprises 3 online courses: Foundations in Adolescent Behavioural Health; Translational Research Methods; Advanced Research Education. Also comprises mentoring and 3 service-learning modules focussed on designing, implementing and evaluating interventions in the community. CBI also involved developing/implementing an idea for a D&I project as part of the training.	Post
Carlfjord, 2017 [47]	Linköping University, Sweden	7. Implementation Theory and Practice	Course/F-F^a^	Doctoral-level course on implementation science with an emphasis on implementation theories, models and frameworks. CBI is held between Sept-Dec.	Doct
Farrell, 2014A [90]	National Cancer Institute, USA	8. Research to Reality cyber Seminars	Seminars/remote	This CBI and the two listed below (Farrell B and Farrell C) are part of an online community of practice providing training and support in D&I training, broken down into distinct CBIs. The cyber series (described here) is a 10-month cycle of seminars (individuals can choose which to attend), comprising web-based presentations focussed on translating research into practice and pairing researchers with practitioners to conduct implementation research.	NR
Farrell, 2014B [90]	National Cancer Institute, USA	9. Research to Reality Mentorship Program	Mentorship programme/remote	Pilot mentorship programme where (through a website) individuals follow 6 mentor-mentee pairs through monthly storyboards (not clear for how many months) that highlight their progress in implementing evidence-based practices in their communities.	NR
Farrell, 2014C [90]	National Cancer Institute, USA	10. Advanced Topics in Implementation Science	Webinars/remote	An online platform that convenes implementation scientists to teach and share perspectives on current implementation science topics and their implementation research and practice.	NR
Gonzalez, 2012A [104]	University of California, USA	11. Translating Evidence into Practice – Implementation & Dissemination courses	Modules integrated in a master’s programme/F-F	A series of modules that have been integrated into the Training in Clinical Research (TICR) programme, including the 2-year master’s programme in clinical research - comprises 5 courses related to translating evidence into practice with a focus on theory and design, individual and system-level implementation strategies and community-engaged research and policy. Three of the 5 courses must be selected.	Post
Gonzalez, 2012B [104]	University of California, USA	12. Implementation & Dissemination Training	Course/NR	Independent of the master’s programme (described in Gonzalez 2012A)—intensive training experience where the principles of implementation and dissemination science are applied through participation in a quality improvement, delivery system innovation or health promotion project.	NR
Gonzalez, 2012C [104]	University of California, USA	13. Implementation & Dissemination Science Grant Writing Course	Course/NR	This course is listed in Table 1 of the article as one of the relevant activities offered as part of the TICR that relates to D&I but is not described in any detail elsewhere in the paper.	NR
Goodenough, 2013 [49]	Dementia Research Collaborative, Australia	14. Knowledge Translation	Workshop/F-F	One-day workshop on knowledge translation (KT) in dementia which also includes 3 optional seminars of which delegates choose two—2 seminars are related to clinical updates in dementia and the third relates to principles of KT (2-h seminar). The article focusses mainly on the seminar which is part of the workshop and how KT knowledge and practice differ between those that did and did not opt to attend the seminar.	NR
Greenhalgh, 2006 [105]	University College London, UK	15. Masters in Primary Health Care	Modules integrated in a master’s programme/remote	This MSc includes a module on ‘Getting research into practice’—the name of the module implies that it has overall relevance to D&I but the specifics of this is not provided in the article. The whole MSc is structured around study units that run on a 7-week cycle and assume 10 h a week of student input.	Post
Jones, 2015 [91]	University of Melbourne, Australia	16. Knowledge Translation for Researchers	Course/F-F	One day course (pilot course was half-day) on practical KT strategies for researchers across 5 themes—theory, planning KT, developing relationships, communicating research and evaluating KT impact.	NR
Kho, 2009 [106]	Canadian Institute for Health Research (CIHR), Canada	17. Knowledge Translation Summer Institute (KTSI)	Training institute/F-F	Four-day intensive CBI that focusses on health services, policy, population and public health areas. Delegates engage in activities relating to planning/carrying out KT research, stakeholder engagement, KT concepts, methods and theories. CBI includes a mentoring component. **This CBI is also described in Leung, 2010 and is linked to Straus, 2011C.**	Doct/PD
Leung, 2010 [107]	CIHR, Canada	17. End-of Grant KT Plan (part of the KTSI described above)	Course integrated into a training institute/F-F	This course formed part of the 4-day KT summer institute previously described **(see Kho, 2009: also linked to Straus, 2011C).** Trainees are assigned to small groups to work on case studies for developing an end-of-grant KT plan.	**Doct**
Luke, 2016 [92]	Washington University, USA	2. Implementation Research Institute	Training institute/blended	Two-year CBI comprising 2 annual week-long training sessions in implementation science in mental health, field implementation projects and attending implementation science conferences and mentorship (monthly mentoring call). This article specifically examines the benefits of the mentoring. **CBI also discussed in Baumann, 2019, Brownson, 2017A and Proctor, 2013.**	**Doct**
Marriott, 2016 [93]	Society for Implementation Research Collaboration (SIRC), USA	18. Implementation Development Workshop	Workshop/F-F or remote	CBI is provided in F-F format (6 h) and online (2 h). Provides individuals with the opportunity to vet projects and get feedback at the proposal stage of their implementation research (only open to members of the Network of Expertise). There is an opportunity for those interested in presenting their project ideas to do so but this is not a requirement. This article compares delegates’ attitudes towards the F-F versus remote format.	NR
Means, 2016 [94]	University of Washington, USA	19. Implementation Science and Health Metrics	PhD/NR	CBI focusses on the technical and applied skills to bridge the ‘know-do’ gap. Draws from multiple disciplines including epidemiology, biostatistics, health services research, economics and anthropology.	Doct
Meissner, 2013 [48]	National Institute for Health, USA	20. Training in Dissemination and Implementation Research in Health (TIDIRH)	Training Institute/F-F	Five-day CBI aimed at preparing investigators to conduct implementation research to increase the submission rate and quality of D&I grant applications and publications by returning to their home institute and teaching others what they have learnt. CBI also involves developing an idea for a D&I project as part of the training. **CBI also discussed in Vinson, 2019.**	PD
Moore, 2018 [58]	Knowledge Institute, St Michael’s Hospital, USA	21. Practising Knowledge Translation	Course/blended	Six-month CBI which focusses on the use of theories, models and frameworks and knowledge, skills and self-efficacy in KT intervention development and implementation – incorporates behaviour change theories, frameworks and evaluation cycles. It involves developing an idea for a D&I project as part of the training – comprises a 3-day workshop and 11 webinars over the 6-month period.	NR
Moore, 2013 [108]	Vanderbilt University, USA	22. EBP 11: Evaluating and Applying Evidence	Course that is integrated in a Doctor of Nursing Program (DNP)/blended	Offered as part of the first year of the Doctor of Nursing Program. Covers various content including knowledge translation in complex health care systems. The course runs over one term with three 2-h sessions in the first week and weekly sessions thereafter.	Doct
Morrato, 2015 [95]	University of Colorado, USA	23. Introduction to Dissemination and Implementation	Workshop/F-F^b^	Introductory workshop focussed on concepts and methods in D&I such as design, theory, implementation strategies, measurement and tools and resources. The workshop is 1.5 days with the second (half-day) optional.	Post
Norton, 2014 [96]	University of Alabama, USA	24. Dissemination and Implementation in Health	Course/blended	CBI that is spread over one term (course participants meet twice weekly for 75 min and students and researchers meet once weekly for 1 h throughout). Aimed at students and academic researchers. For students, it involves a 3-credit course offered as an elective comprising didactic lectures and classroom activities including viewing online and audio-recorded presentations. Students are paired with academic researchers to work on a ‘collaborative project’ on developing and implementing an evidence-based practice.	Post
Osanjo, 2016 [97]	University of Nairobi, Kenya	25. Implementation Science Fellowship Program	Fellowship programme/blended	A two-year CBI with one substantive time commitment of 3-months of didactic training at the beginning of the program with the rest completed online, and in evenings/weekends. Focussed on all key elements of implementation science including research methods, developing, implementing, evaluating and sustaining interventions and stakeholder engagement. CBI involves undertaking a research project.	Post
Padek, 2018 [98]	Washington University, USA	3. The Mentored Training for Dissemination and Implementation Research in Cancer (MT-DIRC)	Training program/blended	Two-year CBI comprising a 5-day long intensive training institute held once a year, that individuals attend twice (once each year), mentorship throughout on their research ideas, calls to fellows and webinar sessions. Also involves working on a research proposal or project. **CBI also discussed in Brownson, 2017B.**	PD
Park, 2018 [99]	Knowledge Institute, St Michaels Hospital, USA	26. Foundations in Knowledge Translation	Training initiative/blended	Two-year CBI which provides team training in KT practice and includes three tailored workshops, 2 years of coaching and an online platform for training materials and knowledge exchange. Topics included KT funding, evaluation and sustainability. CBI involved applying in teams for the training.	NR
Proctor, 2013 [109]	Washington University, USA	2. Implementation Research Institute	Training institute/blended	Two-year CBI comprising a 5-day long intensive training institute in implementation science in mental health that is held once a year (individuals attend it twice; once each year), field implementation projects and attending implementation science conferences, plus mentoring throughout. Also involves working on a research proposal or project. **CBI also discussed in Baumann, 2019, Brownson, 2017A and Luke, 2016.**	Doct
Proctor, 2019 [100]	Washington University, USA	27. Training in Implementation Practice Leadership (TRIPLE)	Training programme/F-F^c^	CBI Comprises lectures, group exercises and reading and involved optional conference calls for more in-depth coaching and mentoring. Trainees develop and trial a small-scale implementation project in their setting. Content focusses on knowledge and skills necessary to ‘lead’ the implementation and evaluation of evidence-based practices. CBI is spread over 3 months approximately with 3 half-days, 4 weeks apart.	NR
Ramaswamy, 2019 [62]	Chapel Hill, University of North Carolina, USA	28. Applied Implementation Science	Module that is integrated into a master’s programme/remote	Umbrella term for a 4-course sequence in implementation science (on design, implementation, improvement and evaluation) which is part of the masters in public health and open to doctoral students and students in other Schools. Each module is approximately 1–2 weeks in duration (full duration of course is not reported).	Post
Riner, 2015 [101]	Indiana university, USA	29. Evidence-based Research and Translational Science: Inquiry 11	Course that is integrated into a DNP/F-F	Part of the Doctor of Nursing Practice that comprises two didactic courses (Inquiry 1 and 11) and seven practicum credits. Inquiry 1 involves a literature review of a best practice. Inquiry 2 involves implementing the best practice (and is the focus of this article) with the practicum providing the framework for the project. Inquiry 11 is made of up 4 modules related to planning and implementing a project.	Doct
Straus, 2011A [110]	CIHR, Canada	30. Knowledge Translation Seminars (stream 1)	Seminar/remote	Article describes a number of CBIs relating to different KT ‘streams’ (1,2,3)—each stream is directed towards different groups of individuals. Within each stream, there are several distinct CBIs. Stream 1 focussses on advanced training in the science and practice of KT—the breakdown of these CBIs is described here (see Straus 2011A-2011G). This CBI is a monthly seminar series that focusses on KT methodology	Post
Straus, 2011B [110]	CIHR, Canada	31. Research Operations (stream 1)	Seminar/remote	CBI involves quarterly seminars focussed on preparing, writing & reviewing grants, ethics submissions and manuscripts.	Post
Straus, 2011C [110]	CIHR, Canada	17. Knowledge Translation Summer Institute (KTSI) (stream 1)	Training institute/F-F	CBI is a training institute that is focussed on a different theme each year—e.g. exploring the knowledge to action framework or developing KT interventions—and addresses one or more of the KT core competencies. A mentoring component is included. **CBI also described in Kho, 2009 and Leung, 2010.**	Post
Straus, 2011D [110]	CIHR, Canada	32. Introduction to Evidence-based Medicine (stream 1)	Course/NR	CBI mentioned in the text and highlighted in Table 2 of the article, but no further information provided.	Post
Straus, 2011E [110]	CIHR, Canada	33. Introduction to Systematic Reviews (stream 1)	Course/remote or F-F	CBI mentioned in the text and highlighted in Table 2 of the article, but no further information provided.	Post
Straus, 2011F [110]	CIHR, Canada	34. Pragmatic KT Trials (Stream 1)	Course/remote or F-F	CBI mentioned in the text and highlighted in Table 2 of the article, but no further information provided.	Post
Straus, 2011G [110]	CIHR, Canada	35. End of Grant KT (Stream 1)	Course/NR	Aimed at helping trainees as they prepare grants. Please note an ‘end of grant’ session is integrated in the KTSI (described in the KTSI in Straus 2011C), but this course is also available as a standalone ‘CBI’, i.e., separate to the KTSI, so is considered as a separate entry here. **The content/focus of this CBI is linked to Kho, 2009 and Straus 2011C**.	Post
Straus, 2011H [110]	CIHR, Canada	36. Integrated KT (Stream 2)	Course/F-F or remote	CBI in Stream 2 of the training which provides training in the basic principles of KT. Modular integrated KT course, reflecting the knowledge to action loop (no further information provided).	NR
Straus, 2011I [110]	CIHR, Canada	37. End of Grant KT (Stream 2)	Course/F-F^d^	One-day CBI aimed at helping trainees as they prepare grants. Please note an ‘end of grant’ session is integrated in the KTSI (described in the KTSI in Straus 2011C), but this course is also available as a standalone ‘CBI’, i.e. separate to the KTSI, so it considered as a separate entry here. **The content/focus of this CBI is linked to Kho, 2009 and Straus 2011C.**	NR
Straus, 2011J [110]	CIHR, Canada	38. Introduction to KT (Stream 3)	Course/NR	CBI which is part of Stream 3 which focusses on basic training in KT for knowledge users. This CBI provides an overview of KT principles (no further information on it is provided).	NR
Straus, 2011K [110]	CIHR, Canada	39. Basics of KT (Stream 3)	Course/F-F	CBI covers the basics of KT principles and the opportunity for students to apply these to a project in their own setting.	NR
Ulrich, 2017 [102]	University of Heidelberg, Germany	40. Master of Science in Health Service Research and Implementation Science	Modules that are part of a master’s program/F-F	Two-year master’s program made up of five streams. One stream comprises 4 implementation science-related courses on concepts and methods; quality improvement and evaluation; organisational development in healthcare; putting research findings into practice (the latter involves writing an outline for an implementation science-related project).	Post
Vinson, 2019 [103]	National Institute for Health, USA	20. Training in Dissemination and Implementation Research in Health (TIDIRH)	Training institute/F-F^e^	Five-day CBI aimed at preparing investigators to conduct implementation research to increase the submission rate and quality of D&I grant applications and publications by returning to their home institute and teaching others what they have learnt. CBI also involved developing an idea for a D&I project as part of the training. **CBI also discussed in Meissner, 2013.**	**PD**
Wahabi, 2011 [111]	King Saud University, Saudi Arabia	41. Advanced Evidence-Based Healthcare	Workshop/F-F	Train the trainer’s workshop to help enhance attendees’ abilities as clinical tutors in evidence-based medicine. Focussed on two things: 1) debate - various topics including, key concepts and methods of KT, KT frameworks and barriers and facilitators; 2) planning a KT project.	NR

Text in bold denotes relevant information about the CBI that was not described in the article but was described in another article in the review that focussed on the same CBI—the linked article(s) are highlighted in bold in the ‘description’ column

Where articles are numbered ‘A’, ‘B’ (e.g. Brownson, 2017A, 2017B), these are CBIs that are discussed in the same article which are distinct from one another so are included as separate entries in the table. Each CBI is given a unique identifier to show the findings relating to each CBI

The level of detail in Table 1 varies depending on what was reported in the article. Cells that are coded as ‘NR’ = when information is not reported or not clear

The level of education reported in Table 1 is the minimum level of education the CBI is aimed at. The key is as follows: UG = undergraduate level; PG = postgraduate level; Doct = Doctoral-level; PD = postdoctoral level; NR = not explicitly reported

Notes on mode of delivery column: (a) Carlfjord, 2017—a web-based version of the CBI was delivered in 2014 with two on-site visits; (b) Morrato, 2015—an interactive online e-book was made available for participants as a take home resource; (c) Proctor, 2019—optional conference calls to provide extra support were offered to those individuals that wanted it; (d) Straus, 2011I—article states they are working on developing an online module; (e) Vinson, 2019—webinar sessions were delivered on 2 days for the 2014 and 2015 occurrence of TIDIRH

^a^The ‘type’ of D&I CBI has been defined as the way in which the author(s) of each of the included articles refer to their CBI in the article

**Table 3:** Additional characteristics of the D&I CBIs included in the review

First author/date	Context/profession	Information provided on CBI content and structure^a^	Numbers attending the CBI and evaluation of key findings^b^
Ackerman, 2016 [88]	Cardiology/medical students	Description in text and table on programme curriculum	**1. Action Research Program (ARP)** Six students were selected, with an additional 2 joining in month 7 of the programme (for these 2students training focussed on the experiential clinic-based learning component). The CBI was evaluated through interviews with students and clinicians. Students reported increased understanding of how care delivery systems work, improved clinical skills and confidence in interactions with patients. Clinicians reported increased efficiency at the clinic level and improved job satisfaction as a result of their mentoring role. With regards to the improvement projects, although some ideas were implemented, most did not move from conceptualisation because students did not have enough time dedicated to conduct the project (reported by clinicians) or were not given enough guidance by faculty (reported by students).
Baumann, 2019 [57] (e-print ahead of 2020 publication)	Mental health/multiple	Description in text and reference to another publication on the CBI	**2. Implementation Research Institute (IRI)** Article evaluated the CBI across 4 different cohorts from 2010 to 2013—the first 3 years had 10 delegates and the 4thyear had 12 (43 in total). Applicants selected for the IRI training, versus those that were not, were 6 times more likely to be awarded a D&I grant after attending the IRI, even when controlling for other variables. Applicants’ odds of publishing in the journal ‘Implementation Science’ were higher for earlier alumni, starting at 12% after 1 year out of training to 94% for those 4 years from training (versus non-selected applicants which remained relatively stable). **CBI also described in Brownson, 2017A, Luke, 2016 and Proctor, 2013.**
Brownson, 2017A [18]	Mental health/**multiple**	Brief description (1 paragraph) and references to other publications on the CBI and a website on the CBI	**2. Implementation Research Institute (IRI)** Article states that IRI has trained 43 fellows at the time of publication—the breakdown of numbers of each year is referred to in Baumann, 2019 (above). No evaluative data is included in this article. **CBI also described in Baumann, 2019, Luke, 2016 and Proctor, 2013.**
Brownson, 2017B [18]	Cancer/m**ultiple**	Brief description (1 paragraph) and references to other publications on the CBI and a website on the CBI	**3. Mentored Training for Dissemination & Implementation Research in Cancer (MT-DIRC)** Article states that 14 fellows per year attend the training. CBI not evaluated in this article. **CBI also described in Padek, 2018**.
Brownson, 2017C [18]	NR/NR	Mentioned briefly in the text (< 1 paragraph)	**4. Introduction to D&I Science** No evaluative data reported.
Brownson, 2017D [18]	NR/NR	Mentioned briefly in the text (< 1 paragraph)	**5 Implementing and Evaluating Evidence-based Practice** No evaluative data reported.
Burton, 2016 [89]	Child and adolescent behavioural health/multiple	Description in text, figure on programme plan by semester, and a table of the benefits of a blended theoretical approach	**6. The Institute for Translational Research in Adolescent Behavioural Health** In total 28 scholars were recruited in the first 2 years. Preliminary results from surveys revealed that gaining research experience through real-world service-learning opportunities was a key factor in the decision to apply for the graduate certificate. The online method for presenting coursework proved difficult and required additional time and effort from faculty to help navigate technology. Academic mentors felt the design of the program was beneficial but that they needed more guidance on their role as mentors and the scope of the projects.
Carlfjord, 2017 [47]	Non-specific/multiple	Description in text and tables on the topics/lectures in the curriculum, group discussions and seminars	**7. Implementation Theory and Practice** This CBI occurs once a year and was evaluated over a 5-year period (2011–2015). In total, 101 completed the course, with numbers ranging from 20 to 25 over the years (this is now capped at 20 for practical reasons). Students rated their overall perception of the course and its contents highly. The majority reported the course had contributed to their current knowledge in implementation science and two-thirds felt that the knowledge gained would be very relevant to their work. Additional data collected a few months after course completion revealed that most individuals felt they had used the knowledge gained in their work and that this had been valuable.
Farrell, 2014A [90]	Cancer/multiple	Brief description (1 paragraph) and link to website relating to CBI	**8. Research to Reality Cyber Seminars** Article reports that registrants and participants on the seminars have significantly grown. The first web-seminar was conducted in January 2010 with over 1100 registrants and 700 participants. Since the first webinar through December 2013, nearly 20,000 people have registered for the yearly, 10-month seminar schedule. On average there are 675 registrants and 260 participants each month.
Farrell, 2014B [90]	Cancer/multiple	Brief description (1 paragraph) and link to website relating to CBI	**9. Research to Reality Mentorship Program** No evaluative data reported.
Farrell, 2014C [90]	Cancer/multiple	Brief description (1 paragraph) and link to website relating to CBI	**10. Advanced Topics in Implementation Science** No evaluative data reported.
Gonzalez, 2012A [104]	Non-specific/multiple	Description in text, and online supplementary file (case study for the CBI)	**11. Translating Evidence into Practice – Implementation & Dissemination courses** No evaluative data reported but article states that approximately twenty scholars participated substantively in the IDS curriculum (completing multiple IDS-specific courses and initiating IDS research projects).
Gonzalez, 2012B [104]	Non-specific/multiple	Brief description (1 paragraph) in text	**12. Implementation & Dissemination Training** No evaluative data reported.
Gonzalez, 2012C [104]	Non-specific/multiple	Highlighted as a relevant CBI relating to D&I in a table	**13. IDS Grant Writing Course** No evaluative data reported.
Goodenough, 2013 [49]	Dementia/multiple	Description in text, table of content and learning objectives and link to a website	**14. Knowledge Translation Workshop** This was a one-day workshop on KT in dementia but also included a seminar on KT methods and practices (which the article focusses on). Article states the response rate for the evaluation survey but does not state the number of delegates that attended the KT seminar. Delegates were emailed a survey 6-months post-workshop. Results were compared between those that did and did not opt for the KT seminar as part of the workshop. The KT group reported the highest median number of overall uses of workshop information in daily practice when compared to those that only participated in the clinical seminars - 7.5 vs. 6 (*p* > 0.05). There was a correlation (*p* < 0.05) between the total number of ‘kinds of research use’ (e.g. changed a practice, changed your beliefs) and individual mean scores (average across 5 items) for conceptual research uses (e.g. ‘gave you knowledge on how to care for residents’), and this was stronger for those that attended the KT seminar. Three items stood out—changing a practice, changing a procedure and creating a new policy/guideline. Six separate one-day workshops were held in total.
Greenhalgh, 2006 [105]	Primary health/multiple	Description in text	**15. Getting Research into Practice and Policy** Article briefly summarises students’ evaluations—students highlighted that the online environment provided the opportunity to rehearse and modify potential implementation scenarios of knowledge into practice, the asynchronous nature of the virtual discussions (vs. synchronous) provides more opportunity for reflection and the record of text messages means they have a permanent record of information to refer back to.
Jones, 2015 [91]	Public health/multiple	Brief description in text (1 paragraph)	**16. Knowledge Translation for Researchers** A pilot half-day course was delivered and evaluated prior to the 1-day course being developed (the 1-day course has been delivered twice). No data is provided but the article states that the course has been well received and is relevant and useful to a range of researchers.
Kho, 2009 [106]	Non-specific/multiple	Description in text, table summary of the curriculum, appendix on the small group task, online supplementary files on daily programme and curriculum and small group project and slide deck and responses to essay questions	**17. Knowledge Translation Summer Institute** In total, 30 applicants were offered a place on the training. Article focusses more on ‘lessons learnt’ than evaluative data *per se* but states the CBI provided many invaluable opportunities for attendees, in that all attendees expressed an interest in maintaining relationships, being updated with each other’s work and participating in future training opportunities. **This CBI is also described in Leung, 2010 and is linked to Straus, 2011C.**
Leung, 2010 [107]	Non-specific/**multiple**	Description in text, reference to a publication, appendices on an overview of the CBI, description of the case study, end-of-grant KT plan and guiding questions for group discussions	**17. End-of Grant KT Plan (part of KTSI above)** Article focussses mainly on challenges and recommendations of end-of-grant KT plans. Feedback from attendees and KT experts was that the session was too complex for what would be a small component of the grant proposal. However, the KT experts also emphasised the importance of including a KT component in the grant to increase the likelihood of a successful grant application **(see Kho, 2009 and Straus, 2011C).**
Luke, 2016 [92]	Mental health/**multiple**	Description in text and reference to another publication	**2. Implementation Research Institute** Article reports that 43 fellows in four cohorts have been trained (the breakdown of numbers in each cohort is reported in Baumann, 2019). This article focusses on the mentoring component of the CBI. Mentoring was positively and significantly related to having scientific collaboration 2 years later, including new research, grant submissions and publications. For every additional mentoring relationship that was established, the likelihood of scientific collaboration increased by nearly 7%. **CBI also discussed in Brownson, 2017A, Luke, 2016 and Proctor, 2013.**
Marriott, 2016 [93]	Non-specific/NR	Description in text	**18. Implementation Development Workshops** Between 2011 and 2015, 72 members participated in at least one workshop (number of attendees in each workshop is not clear). 40 participated in face-to-face only, 16 in virtual only, and 16 in both formats. The focus of the article was to compare F-F vs. virtual format for implementation science training. Both were found to be equally acceptable and were effective for collaboration and growth and success in obtaining grants. A third of presenters received funding for their proposals and more than 80% of presenters said they would present again.
Means, 2016 [94]	Global health/NR	Brief description (1 paragraph)	**19. Implementation Science and Health Metrics** No evaluative data provided.
Meissner, 2013 [48]	Non-specific/multiple	Description in text and list of faculty and daily curriculum	**20. Training in Dissemination and Implementation Research in Health (TIDIRH)** Thirty-five applicants were accepted on the course. Attendees rated CBI as ‘very helpful’. A 6-month follow-up survey (97% response rate) revealed 72% had initiated a new grant proposal, 28% had received funding and 77% had used skills from TIDIRH to influence peers about dissemination & implementation research, build research networks, organise presentations and teaching and lead interdisciplinary teams to conduct D&I research. **CBI also discussed in Vinson, 2019.**
Moore, 2018 [58]	Non-specific/ Multiple	Description in text, link to a website, table of course structure and delivery and online supplementary file on core competencies	**21. Practising Knowledge Translation** Seventeen participants were enrolled on the course. Data were collected at 3, 6 and 12 months. Attendees reported significant positive effects in terms of—increased use of implementing theories, models and frameworks and increased knowledge of developing evidence-informed programmes, evidence implementation, evidence evaluation, sustainability scale and spread and context assessment (with self-efficacy increasing across these measures too).
Moore, 2013 [108]	Nursing/nurses	Description in text and table of competencies	**22. EBP 11: Evaluating and Applying Evidence** Numbers on the course have increased steadily from 2009 (32) to 2013 (64). No specific evaluation data relating to the EBP 11 module was provided. Students rated the overall CBI highly and identified several strengths, including – exposure to different research article critique instruments and group interactions.
Morrato, 2015 [95]	Non-specific/multiple	Description in text, link to website, tables on agenda, faculty and D&I CBI resources	**23. Introduction to Dissemination and Implementation** Sixty-eight delegates attended day one and 11 also attended the half-day on day two (which was optional). Data collected 1 week after the CBI (from 34/68 responses) revealed that: 100% ‘strongly agreed’ they were satisfied with the training and 97% felt the workbook was a valuable resource. Delegates that attended the day 2 mentoring session ‘strongly agreed’ that working closely with faculty/experts increased their confidence. At 6-month follow-up, evidence of 23 new manuscripts and grant proposals were found.
Norton, 2014 [96]	Public health/multiple	Description in text and a table of weekly topics	**24. Dissemination and Implementation in Health** A total of 24 students enrolled in the course and 19 faculty researchers participated. Students strongly agreed that they would recommend the course to other students, they enjoyed it and were able to apply what they learnt to their D&I project. Faculty rated it highly too and strongly agreed that they would recommend participation in the course to other faculty and were interested in learning about D&I from students. The collaborative learning projects were rated by both as one of the most valuable aspects. Suggestions for improvement centred on (for students) course logistics, more meetings to discuss collaborative project, more time from start of course to when they meet faculty partners. Faculty reported the need for clearer expectations for the collaborative learning project and the opportunity to attend lectures.
Osanjo, 2016 [97]	Non-specific/multiple	Description in text and a table on curriculum	**25. Implementation Science Fellowship Program** There were 5 trainees in the two cohorts that undertook the course. A survey (in years 1 and 2) revealed a high degree of satisfaction with most aspect of the CBI including content, duration and attachment sites. Fellows expressed high satisfaction with the mentorship program and would prefer the existing mentorship arrangement to be extended. Some fellows indicated they were already applying the skills gained at their home institutions. Fellows have embarked on PhD programmes in dissemination and implementation (*N* = 4), secured funding (*N* = 3) and most (85%) identify implementation science as a component of their work activity.
Padek, 2018 [98]	Cancer/multiple	Description in text, tables on faculty and mentoring, weblink to training, and an online supplementary file on the agenda	**3. The Mentored Training for Dissemination and Implementation Research in Cancer (MT-DIRC)** On average 14 fellows attend the training each year and from 2014 to2017, 56 fellows have participated. Forty-three dissemination and implementation science competencies were assessed—all improved from baseline to 6 months and 18 months. The effect was apparent across beginner, intermediate, and advanced fellows. Mentoring was rated very highly by fellows (and more highly than by the mentors). The importance of different mentoring activities was linked to fellows’ satisfaction with the mentoring activities. **CBI also discussed in Brownson, 2017B.**
Park, 2018 [99]	Non-specific/multiple	Description in text, and an online supplementary file on the agenda	**26. Foundations in Knowledge Translation** A total of 46 participants across two cohorts have completed the training (16 teams ranging in size from 2–4 people). Surveys (at 6, 12, 18, 24 months) revealed attendees’ self-efficacy in evidence-based practices, KT activities, and using evidence to inform practice increased over time. Focus groups and interviews indicated that confidence in using KT increased from baseline to 24 months and that training helped to achieve attendees’ KT objectives, plan their projects and solve problems. Teams with high self-reported capacity and commitment to implement projects and ‘buy-in’ from upper management that resulted in securing funding and resources were stated as important to achieve goals. Sustained spread of KT practice was observed with 5 teams at 24 months.
Proctor, 2013 [109]	Mental health/multiple	Description in text	**2. Implementation Research Institute (IRI)** Article states that 10 fellows are selected each year. Fellows were very satisfied with the program and would recommend it to colleagues. Fellows and faculty rated the calibre of their counterparts as ‘excellent’. Fellows from the first 3 cohorts have submitted 74 proposals (52 funded) and are beginning to serve as mentors for more junior investigators. A total of 208 publications have been submitted/published (7.64 per fellow) as well as conference presentations and teaching. **CBI also discussed in Baumann, 2019, Brownson, 2017A and Luke, 2016.**
Proctor, 2019 [100]	Behavioural health/multiple	Description in text and table on curriculum	**27. Training in Implementation Practice Leadership (TRIPLE)** Sixteen mid-level leaders were enrolled in the training. Most attendees reported increased implementation leadership skills (86%) and implementation climate (79%) after the training (*p* < 0.05). Implementation leadership skills improved most on the proactive and knowledgeable subscales. For implementation climate, educational support and recognition for using evidence-based practice revealed the greatest increase (post training). Attendees found the training highly acceptable and appropriate and qualitative results indicated that training led to increased organisational implementation as well as leadership skills for attendees.
Ramaswamy, 2019 [62]	Public health/multiple	Description in text, table on courses and descriptions and an online supplementary files on course syllabi, alignment of CBI with competencies	**28. Applied Implementation Science** As of April 2018, a total of 11 sections of the course have been offered, with a total enrolment of 142 (127 of whom were MPH students). Taking the 4 courses collectively, students’ qualitative feedback was positive (e.g. *‘useful tools for the application of implementation science’, ‘practical and allows you to build real skills’*). The degree to which students had applied what they had learned was supported by 8 students embarking on practicums, masters papers and other implementation science-related learning projects.
Riner, 2015 [101]	Nursing/nurses	Description in text and table of competencies	**29. Evidence-based Research and Translational Science: Inquiry 11** No evaluative data provided.
Straus, 2011A [110]	Non-specific/multiple	Brief description (1 paragraph), table of topics and a link to a website	**30. Knowledge Translation Seminars (stream 1)** No evaluative data provided.
Straus, 2011B [110]	Non-specific/multiple	Brief description (1 paragraph) and a link to a website	**31. Research Operations (stream 1)** No evaluative data provided.
Straus, 2011C [110]	Non-specific/multiple	Description in text and a table of themes for CBI	**17. Knowledge Translation Summer Institute (stream 1)** To date (2011) three summer institutes have been held with 90 trainees in total. No specific evaluative data from trainees but article reports that trainees have been involved in 3 publications, the preparation of collaborative multi-site grants and projects and have worked together on education modules and presentations. **CBI also described in Kho, 2009 and linked to Leung, 2010.**
Straus, 2011D [110]	Non-specific/multiple	Brief description (< a paragraph)	**32. Introduction to Evidence-based Medicine (stream 1)** No evaluative data provided.
Straus, 2011E [110]	Non-specific/multiple	Brief description (< a paragraph)	**33. Introduction to Systematic Reviews (stream 1)** No evaluative data provided.
Straus, 2011F [110]	Non-specific/multiple	Brief description (< a paragraph) )	**34. Pragmatic KT Trials (stream 1)** No evaluative data provided.
Straus, 2011G [110]	Non-specific/multiple	Brief description (1 paragraph) and a link to website	**35. End of Grant KT (Stream 1)** No evaluative data provided. **This CBI is linked to Kho, 2009 and Straus 2011C**,
Straus, 2011H [110]	Non-specific/multiple	Brief description (1 paragraph) and a link to website	**36. Integrated KT (stream 2)** No evaluative data provided.
Straus, 2011I [110]	Non-specific/multiple	Brief description (1 paragraph)	**37. End of Grant KT (Stream 2)** No evaluative data provided.
Straus, 2011J [110]	Non-specific/multiple	Brief description (1 paragraph)	**38. Introduction to KT (Stream 3)** No evaluative data provided.
Straus, 2011K [110]	Non-specific/multiple	Brief description (1 paragraph)	**39. Basics of KT (stream 3)** Article does not provide evaluative data but does state that this CBI has been held on two occasions including colleagues from 16 teams.
Ulrich, 2017 [102]	Non-specific/multiple	Description in text, table of curriculum, website link to MSc module manual	**40. Master of Science in Health Service Research and Implementation Science** The first cohort of students had 13 students, and the second cohort had 23 students. Article provides data on expectations (from the perspective of students, experts and teaching staff) of what should be included in the course, rather than their evaluations of the course per se. 27/42 of the competencies were felt to be crucial or very important by more than 80% of participants. 6/8 items that individuals rated as very important specifically related to implementation in practice were in this category, e.g. knowledge of implementation strategies and barriers and enablers to implementation.
Vinson, 2019 [103]	Non-specific/**multiple**	Description in text, table of course content, reference to another publication on the CBI	**20. Training in Dissemination and Implementation Research in Health (TIDIRH)** In total 197 trainees have undertaken the training between 2011-2015. Article evaluated long-term impact on trainees that attended one of the TIDIRH’s over a 5-year period (TIDIRH held once annually). Selected applicants were compared to unselected applicants for applications for NIH peer-reviewed funding. A survey of trainees and unselected applicants as well as a faculty survey was conducted. Thirty-eight per cent of trainees stated they had extensive contact with faculty following training and a further 38% indicated they had at least limited contact. Twenty-four per cent had extensive collaborations with other fellows after the training and 43% had at least limited contact. Overall trainees submitted more funding applications than unselected applicants and had better funding outcomes (25% vs. 19%). **CBI also discussed in Meissner, 2013.**
Wahabi, 2011 [111]	Family medicine/medics	Description in text, table of CBI contents, online supplementary files on CBI format and objectives and project objectives	**41. Advanced Evidence-Based Healthcare** Twenty-one participants attended the workshop. Participants indicated that the ‘debate approach’ added a new dimension to their evidence-based medicine skills by adding purpose and motivation but that their performance would have been better if they had been offered a practical demonstration of how to conduct a debate. The KT project enhanced understanding of the relationship between evidence and implementation, however, some maintained this fell out the scope of the role of the doctor.

Text in bold denotes relevant information about the CBI that was not described in the article but was described in another article in the review that focussed on the same CBI—the linked article(s) are highlighted in bold in the ‘description’ column.

Where articles are numbered ‘A’, ‘B’ (e.g. Brownson, 2017A, 2017B), these are CBIs that are discussed in the same article which are distinct from one another so are included as separate entries in the table. Each CBI is given a unique identifier to show which findings refer to each CBI.

Whenever possible, we have provided information on the CBIs—the level of detail in Table 1 varies depending on what was reported in the article. Cells that are coded as ‘NR’ = when information is not explicitly reported.

^a^The types of information provided on the CBI are listed in the table—this is only a high-level summary and should not be used as an indicator of article quality (the content and structure of these CBIs will be examined in follow-up work)

^b^A high-level summary of evaluative findings on the CBI (where reported) is provided—a more detailed analysis will be conducted in follow-up work.

### Eligibility criteria and application process

The majority of CBIs were aimed at individuals who had undertaken or were undertaking a postgraduate qualification (*N* = 12) [62, 89, 95–97, 102, 104^a^, 105, 110^a-g^], and to a lesser extent, doctoral/postdoctoral (*N* = 10 ) [18^a,b^, 47, 48, 57, 92, 94, 98, 101, 103, 106–109] or undergraduate level individuals (*N* = 1) [88]. For the remaining CBIs (*N* = 18 CBIs) [18^c,d^, 49, 58, 90^a-c^, 91, 93, 99, 100, 104^a,b^, 110^i,k^, 111], it was not reported. CBIs were run through academic institutions (*N* = 22) [18^a-d^, 47, 57, 62, 88, 89, 100–102, 104^a-c^, 108, 109, 111, 91–98] or healthcare organisations/institutions working in D&I-related areas (*N* = 17) [48, 58, 90^a-c^, 99, 103, 106, 107, 110^a-k^] and to a lesser extent through D&I-related collaboratives (*N* = 2) [49, 93].

Seventeen of the CBIs focussed the training towards a specific context, including cancer (*N* = 4 ) [18^b^, 90^a-c^, 98], public or global health (*N* = 4) [2, 91, 94, 96], nursing (*N* = 2) [101, 108], behavioural health (2) [89, 100], cardiology (*N* = 1) [88], family medicine (*N* = 1) [111], mental health (*N* = 1) [18^a^, 57, 92, 109], dementia (*N* = 1) [49] and primary care (*N* = 1) [105]. The remaining CBIs were either not restricted to a heath or social care setting (*N* = 22) [47, 48, 58, 93, 95, 97, 99, 102–104^a-c^, 107, 110^a-k^] or it was not reported (*N* = 2 CBIs) [18^c,d^]. Most of the CBIs were aimed at multiple professions(*N* = 33) [18^a,d^, 47–49, 57, 58, 62, 90^a-c^, 91, 92, 95–100, 102–104^a-c^, 105–107, 109, 110^a-k^] with fewer confined to specific groups of individuals, including medical students or medics (*N* = 2) [88, 111] or nurses (*N* = 2) [101, 108]: for the remainder, it was not reported (*N* = 4) [18^c,d^, 93, 94].

Ten of the CBIs provided information on the application and selection process. This ranged from individuals taking a formative assessment to ensure they had the requisite knowledge and skills in evidence-based medicine [111]; providing evidence that they had not received major research funding in D&I research before [48]; writing a 1.5–2 page concept paper describing a D&I research project they would like to undertake as part of the training [48, 57, 98, 109]; detailing prior experience with implementation and/or health science research [48, 57, 58, 102]; producing a cover letter or statement to demonstrate a motivation to undertake the CBI, pursue a career in D&I and/or their long-term research agenda [48, 57, 58, 97, 98, 102, 109]; obtaining a letter of support or character reference from their workplace [48, 97, 98, 109]; providing evidence of academic grades or research productivity [48, 57, 102, 106, 109]; answering essay questions [106]; and undertaking interviews [88, 102]. One CBI also required individuals to apply in a team (i.e. a joint application involving other individuals) whereby they had to explain a D&I-related project they would like to implement in their workplace to address a healthcare-related challenge [99].

Additional data on the competitive nature of the application process was referred to for six of the CBIs. The TIDIRH [48, 103] had a total of 266 investigators applying in the first year (2011) for 30 places [48], with 1100 applicants over a 5-year evaluation period (2011–2015) for 199 places [103], and in 2019, over 200 applicants applied for 50 available training slots [103]. The IRI [18^a^, 57, 92, 109] accepted approximately 10 fellows each year with a total of 31 fellows over the first 3 years (2010–2012) from a total pool of 86 applicants [109], with other data derived across 4 separate occurrences of the IRI training reporting a 43/124 acceptance rate [57, 92]. The KTSI [106, 107, 110^c^] had 150 trainees that applied for 30 places [106] while the MT-DIRC [18^b^, 98] offered 56 fellows a place over the 4 occurrences of the training (2014–2017) [98]. The ‘Action Research Programme’ reported that only 6 students were accepted at the start of the programme’ [88], and numbers on a master’s (in ‘health services research and implementation science’) [102] and a doctoral-level course (on ‘implementation science’) [47] were capped at 20 due to practical reasons, despite the demand for the courses growing [47].

### Content and structure of the CBI

The level of detail on the content and structure of the 41 CBIs included in the review varied considerably (largely due to the differing aims of the included articles). It is beyond the scope of the present review to examine this in detail here, but an individual breakdown of the information supplied relating to the content of the CBI (e.g. weblinks, course handbooks, workshop agendas) can be found in Table 2. While we are not suggesting here that the number of files, tables or supplementary documents each article provides for each CBI should be used as an indicator of the quality of the article, this does serve to illustrate the type of information authors are providing when reporting on D&I CBIs. Further inspection of the content of the CBIs will be explored as part of our larger programme of work on capacity building in D&I.

#### Evaluation and impact of CBIs

Of the 41 CBIs included in the review, evaluative data was provided for 21 CBIs [47–49, 57, 58, 62, 88–90^a^, 91–93, 95–100, 102, 103, 106–110^c^, 111]. We provide here a high-level summary of key themes (see also Table 2).

##### Overall perception

CBIs were rated ‘positively’ by individuals—in terms of the CBI itself and/or the importance of the contents [47, 95, 97, 98, 102, 108], overall satisfaction [95], acceptability and appropriateness [93, 100], usefulness of tools/methods [62], helpfulness [48] and likelihood of recommending the CBI to others [96].

##### Knowledge and skills

Knowledge and use of D&I principles as well as confidence in conducting D&I activities increased as a result of the CBI [47, 49, 58, 95, 97–100, 111]. Individuals reported using D&I skills they acquired as a result of the training to influence and train peers in D&I [48]; be involved in research networks [48]; deliver educational modules and presentations [110^c^], embark on practicums, master’s papers and other projects [62]; and serve as mentors for more junior investigators [109].

##### Project-based work

Conducting a D&I-related project was reported as one of the main reasons for applying for a CBI [89] and one of the most valuable aspects [96] with individuals reporting this helped to enhance their understanding of the relationship between evidence and implementation [111]. However, individuals also raised the need for more time to conduct projects and more guidance from faculty on the scope of the projects [89, 96]—while some project ideas were implemented, most did not move beyond conceptualization due to lack of time or guidance from faculty [88].

##### Research productivity

Undertaking and completing a CBI was related to research productivity in terms of applying for and/or being awarded funding for D&I research [48, 57, 92, 93, 95, 97, 99, 103, 109, 110^c^], writing publications [92, 109, 110^c^] and embarking on D&I-related PhD programmes [97]. Individuals also reported collaborating with other trainees [103] and expressed interest in maintaining relationships and being updated on each other’s work [106] after the completion of training.

## Discussion

To the best of our knowledge, this is the first systematic review of its kind to identify and collate the type and range of D&I CBIs relating to teaching and training that have been described and/or appraised in the academic literature. An array of training opportunities from countries across the world were uncovered, aimed at numerous professions, focussed on different contexts and ranging in delivery format, duration, structure and content.

This review was (in part) in response to an editorial calling for a greater number of publications on the development and evaluation of D&I CBI training initiatives [52]—we took this call one step further by synthesising the collective evidence published to date. Our research has raised several important implications for the development and delivery of future D&I CBIs as well as their reporting, discussion and appraisal in academic journals. Here, we discuss some of the most pertinent overarching challenges we believe should be prioritised in terms of building capacity in teaching and training in D&I and in how these training endeavours are reported and disseminated for wider use.

### Demand and importance of D&I training

While our findings, supported by the wider evidence-base [59, 112], highlight the recognised international demand and importance of D&I CBIs, we also found an unmet need for D&I training. For some CBIs, enrolment may only occur once a year and/or may have strict eligibility criteria (e.g. specific qualifications or experience), which significantly limits the pool of individuals whom are able to apply. The highly selective nature and low reported acceptance rates of some CBIs [48, 57, 92, 98, 103, 109] also suggest that the demand for training from the wider population is likely much higher—oversubscriptions to D&I conferences, meetings and initiatives provide further support for this view [7, 17, 59, 75].

Also, of note is that many of the CBIs we delineated centred on advancing postgraduate or postdoctoral researchers’ D&I skills. While these CBIs were designed specifically for building research capacity, so do not by nature restrict options for other kinds of learners (e.g. practitioners, policy makers), there does appear to be fewer training options for individuals newer to the field, which could widen the gap between novices and those already skilled in D&I [59]. Greater emphasis on reaching out to predoctoral individuals, practitioners, policy makers and consumers [14, 16, 59] and publishing findings on such CBIs after they have been evaluated is required if we are to gain a better understanding of training needs, priorities and challenges from a diverse range of learners. Additional efforts are required to train multidisciplinary teams (not just individuals), whom are often critical to the successful design and execution of implementation research and practice [16, 113, 114] and on delivering training in low resource settings, whom encounter unique challenges in implementing evidence-based practices due to limited financial resources and healthcare workforce [115–117] (only 3 D&I CBIs in our review focussed on this [97, 99, 111]).

### Availability and accessibility of D&I training and resources

Creative approaches to providing support in D&I are required if/when local institutional support is lacking—which may often be the case given the relative infancy of the field [118] and proportionately small pool of experts able to provide senior mentorship [60]. Examples of such creative approaches uncovered in our review include the use of online platforms to provide mentorship support [90^b^], web-networking to enhance research connections and obtain feedback on research activities [48, 90^c^] and webinars/online seminar series [90^a^, 100^a,b^] to share D&I learnings. More widely, a whole host of additional training opportunities and other resources exist (many of which are free)—including interactive web-based tools [63], networks and discussion forums [42], MOOCs and online courses [119–122] and numerous guides on D&I methodologies [123–131].

Preliminary evidence indicates that individuals are not always aware of the existence of D&I resources nor may they be aware how to access them [132, 133]. Arguably, the D&I community may benefit from more focussed efforts of dissemination in order to reach a wider critical mass of individuals interested in learning about D&I—a point which is of particular importance when no other training option is available (e.g. due to cost or time). While some organisations have made steps to providing lists of D&I resources and training opportunities on their websites [134–138], a more general repository [17] where all the up-to-date evidence and training could be logged is likely to be of significant merit to the field.

### Barriers to effective training

Systematic reviews and research in areas related to D&I (e.g. behavioural sciences, quality improvement and patient safety) report that competing educational demands, time, faculty expertise, motivation and institutional culture are important determinants of successful curriculum implementation and/or completion [139–142]. Parallels can be drawn from our review, with lack of time to conduct D&I projects and insufficient guidance on projects being raised as issues by faculty and trainees [88, 89, 96]. More widely in the literature, costs and time constraints are reported as major factors in the decision to undertake knowledge translation training [50, 63], particularly for those from low resources settings [63]. These findings, while only preliminary, highlight the need to examine different systems and individuals in which D&I curriculum will be implemented, alongside the determinants of developing, delivering and accessing curriculum within these systems for a variety of learners. Doing this will better enable strategies to be put in place to overcome barriers to implementation of D&I CBIs and, in turn, help to address the recognised deficit in training opportunities [14, 75, 80, 112].

### Standardisation in reporting

Unlike other areas of research, where reporting guidelines exist, e.g. for systematic reviews [143], implementation research [144] and intervention reporting [87], there is no equivalent resource specific to the reporting of D&I CBIs. Systematic reviews on knowledge translation interventions (a related and synonymous term with implementation science [65–67, 145, 146]) have raised how inconsistencies in intervention reporting hamper evidence synthesis [147–149]. In the same way through this review, we found that variabilities in reporting D&I CBIs (both in terms of describing and evaluating) can make literature synthesis problematic. While this challenge is not surprising given the differing aims and focus of our included articles (and so is by no means a criticism of the articles we included), it nonetheless highlights an important issue. If we are to use articles like these to learn and further build capacity efforts in D&I—a point raised as important by this journal [52]—greater consistency in reporting is required. We consider the importance of standardisation in more detail here by drawing on two key areas: (1) the reporting of the content and structure of D&I CBIs and (2) the reporting of how D&I CBIs are evaluated.

#### The reporting of the content and structure of D&I CBIs

Due to the extensive scope of this work, we were only able to provide a high-level summary of the content and structure of the CBIs in this paper. However, it was clear when undertaking the review that despite evident similarities on content (e.g. covering measurement or theory), different topics were covered to varying degrees and a consistent curriculum, focussed on inter-disciplinary competencies, was not revealed. While initial steps have been taken to reach consensus on D&I curricula expectations and competencies for various learners (both within the CBIs included in our review and more widely [79, 80, 150, 151]), measures and methods are still developing [152–156] and can be difficult to define [65, 67, 156, 157]. Advocating the adoption of a small, common set of terms (which could then also be used when reporting D&I CBIs) is one way in which a better understanding of the evidence-base in D&I could be reached [65, 71, 152, 154, 155]. Progress is already underway in the reporting of some areas of implementation methodology (e.g. implementation outcomes [71], implementation strategies [152, 154], theories, models and frameworks [155]), but we are still a long way off being able to establish a more comprehensive taxonomy of common terms and methods.

#### The reporting of how D&I CBIs are evaluated

Appraising existing CBIs is one way which can help to understand individual needs for D&I research and practice [14, 50, 51, 55] and identify priorities for D&I capacity building [50, 51]. Articles in our review evaluated CBIs to varying degrees. While this would be expected given our eligibility criteria for articles and their subsequent differing aims, without clear and consistent reporting of data, it is difficult to appraise, synthesise and effectively communicate progress in D&I training across different professions, contexts and purposes. Ideally, evaluations of CBIs would be performed on repeated occurrences of the training (to check consistency in the findings across several cohorts) and longitudinally (to assess the longer-term impact of training) to better establish the effectiveness of D&I curricula in enabling desired outcomes in practice (something which few of the articles included in our review did).

In a field where the evidence (and therefore priorities for teaching) is rapidly changing, standardisation in the reporting of key elements of D&I content and structure as well as the evaluation of the CBIs is critical. This understanding and clarity is essential for D&I educators, researchers and implementers to draw meaningful conclusions from the literature on D&I training. In turn, a clearer cumulative assessment of the evidence-base can be reached, so that training successes and challenges as well as educational gaps in the field can be identified and addressed.

### Review caveats

While our review has much to add to the field of capacity building in D&I, several important caveats should be borne in mind when interpreting our results. First, given the extensive scope of our review and its complexity, we were only able to provide a high-level summary of our findings here. We acknowledge that examining the curriculum of each the D&I CBIs may be of interest to the readership of this journal, as may a detailed synthesis of the evaluation of D&I CBIs like those identified in our review. However, while this is something we plan to undertake in future work, it was beyond the scope and aims of the current paper and would not have been possible without significantly compromising on the level and detail of other information required in order to meet our review’s aims. Second, given one of the aims of our review was to show how D&I CBIs are reported in the academic literature and to use these findings to help inform future recommendations on reporting (a point which has been raised as important to explore [52]), we did not include ‘grey literature’ in our review. We are aware of D&I CBIs in the field that have not been written up for publication [158–162] so acknowledge that our review (while intentional) only provides a fieldwide perspective on the academic literature, not the total number of D&I CBIs on offer. Third, to provide a comprehensive account of the literature, we did not exclude articles based on their aims—unsurprisingly, therefore, those that were included differed in focus, ranging from brief or detailed descriptions and/or evaluations of D&I CBIs to general overviews of several initiatives. Fourth, we did not exclude CBIs based on the level of detail authors provided on them. While this was intentional, in order to highlight variabilities in reporting, undoubtedly, this meant that less meaningful conclusions can be drawn from those articles where minimal information was included. Finally, it is worth noting that we examined just one way to build capacity in D&I—providing funding for D&I research networks [163–166], research proposals [68, 167–169], faculty positions and job vacancies [170, 171] and departments and centres [22–36], as well as organising D&I-related conferences and meetings [43–46] are also important avenues for growth.

## Conclusions

This review addresses a clear gap in the evidence-base and helps pave the way for future research on building capacity in D&I. Greater investment in education and training is necessary to increase the cadre of D&I scientists and practitioners. Consistent reporting on D&I CBIs is required to enable greater transparency on the type and range of training opportunities, attitudes towards them and training gaps that need to be prioritised and addressed. Increasing awareness and accessibility to D&I training and resources should also be prioritised. Ultimately, doing this should result in D&I learnings being more clearly communicated so that the best possible D&I CBIs can be developed to achieve the most optimal outcomes. Further work examining the evidence on CBI D&Is (both within the academic literature and more widely) is required.

## Supplementary information

**Additional file 1:.** Data extraction form.

## Data Availability

The datasets are available from the authors on reasonable request.

## Abbreviations

CBI: Capacity building initiative
D&I: Dissemination and implementation science
