# Supplementary material for: Building capacity in dissemination and implementation science: a systematic review of the academic literature on teaching and training initiatives
Source: Implement Sci. 2020 Oct 30;15:97. doi: 10.1186/s13012-020-01051-6 (PMC7597006; doi:10.1186/s13012-020-01051-6)
Supplement: Supplementary file 1 — Additional file 1:. Data extraction form. [file 13012_2020_1051_MOESM1_ESM.docx]

**Supplementary file 1. Data extraction table: Criteria and operational definitions**

| **Criterion** | **Definition of the criterion for the purpose of data extraction** |
| --- | --- |
| **Decision to include article** | In order to proceed with this data extraction form, please confirm that: 1) the author is writing about a D&I CBI that they or their host institution have developed, rather than; 2) reporting on D&I CBI’s in the wider literature (i.e. those the author is aware of but that have been developed and led by other individuals/institutions). If 1 continue with data extraction, if 2 exclude the article. |
| **Criteria pertaining to the key characteristics of the article** | |
| **1. Article date** | The publication date of the article. |
| **2. Lead author** | First author on the article. |
| **3. Organisation** | The lead host organisation. |
| **4. Country** | Country of origin. |
| **Criteria pertaining to the key characteristics of the D&I CBI** | |
| **5. Name of the CBI** | The name of the CBI as defined by the authors. Where a name is not explicitly stated (e.g. the authors state the CBI focuses on implementation science but do not actually give the CBI a specific name), a proxy title should be provided and denoted by the data extractor in italic font. |
| **6. Links to other articles** | If the CBI is the focus of another article, please indicate which article. |
| **7. Part of a larger CBI or standalone** | Indicate whether the CBI is   1. part of a larger CBI - e.g. a module(s) that is integrated into a master’s program   OR   1. a standalone CBI – e.g. a workshop that is a standalone CBI that individuals can sign up for. |
| **8. Educational level** | The minimal level of education that the CBI is aimed at. Please categorise as one of the following:   - Undergraduate - a CBI that is aimed at undergraduate level individuals that fits into one or more of the following criteria: 1) an undergraduate programme in dissemination and implementation science (D&I); 2) part of a undergraduate programme in D&I; 3) available as standalone course in D&I aimed at individuals of an undergraduate level - Postgraduate – a CBI that is aimed at postgraduate level individuals (i.e. those that have completed an undergraduate course) that fits into one or more of the following criteria: 1) a postgraduate programme in D&I; 2)part of a postgraduate programme in D&I; 3) available as standalone course in D&I aimed at individuals of a postgraduate level - Doctoral - a CBI that is aimed at doctoral level individuals that fits into one or more of the following criteria: 1) a doctoral programme in D&I; 2) part of a doctoral programme in D&I; 3) available as a standalone course in D&I aimed at individuals of a doctoral level - Postdoctoral - a CBI that is aimed at postdoctoral level individuals that fits into one or more of the following criteria: 1) a postdoctoral programme in D&I; 2) part of a postdoctoral programme in D&I; 3) available as a standalone course in D&I aimed at individuals of a postdoctoral level - No degree – A CBI aimed at individuals with no degree (at the undergraduate, postgraduate or doctoral level) - Not reported – If it is not clear who the CBI is aimed at or this information is not reported then this should be categorised as ‘NR’ (not reported). This includes those articles where authors do not explicitly state the educational level of the individuals even if they do report their professions (e.g. if the authors state the CBI is aimed at nurses but they do not include the educational level (e.g. ‘undergraduate’, ‘postgraduate’), assumptions should not be made and the criterion should be coded as ‘NR’. |
| **9. What** | The type of D&I CBI (e.g. ‘workshop’, ‘training institute’) as referred to by the author(s) of each of the included articles- e.g. if the author refers to the CBI as a ‘training program’ this is how the CBI should be described in the review.  Please note:   - If an article reports more than one D&I CBI and these are distinct from one another these should be reported separately, e.g. if an organisation describes both a D&I workshop and training institute - No stipulation is placed on the level of detail provided by the authors about a D&I CBI for it to be included, e.g. if the authors only briefly introduce a CBI or highlight the CBI in a table or figure, this is enough for inclusion - The highest level of specificity should be recorded for each type of D&I CBI, i.e. if an article reports on D&I-related modules that form part of a master’s programme, the CBI should be recorded as a ‘master’s programme’ and not as ‘modules’ but it should be acknowledged (where applicable) in the description of the CBI (criteria 13) that it may only be the modules and not the rest of the master’s programme that relate to D&I. |
| **10. Context** | The context in which the CBI is focussed. Please code as follows:   - Non-specific - if a specific context is not mentioned - e.g. the authors state that the CBI is healthcare-focussed - Specific – if the authors state the CBI relates to a specific context – e.g. dementia, cardiology, please note this - Not reported – if it is not reported or not clear whether the CBI is specific or non-specific, please code as ‘NR’ |
| **11. Profession** | The profession(s) that the CBI is aimed at. Please code as follows:   - If the authors specify the CBI is focused towards one profession, please note what this is e.g. - ‘clinicians’, ‘psychologists’ - If the authors specify the CBI is focussed toward multiple professions, please code as ‘multiple’ - If the information is not provided or is not clear, this is denoted as ‘NR’ (not reported). |
| **12. How** | The way in which the CBI is delivered. Please categorise as one of the following:   - F-F - a CBI that is delivered in person only - Remote - a CBI that is not delivered in person but is delivered remotely – e.g. this could be online (either with no person contact or with person contact, e.g., through discussion forums, Skype calls) or over the phone - Blended – a CBI that comprises F-F and remote teaching   Please note, this criterion is specifically interested in how the CBI itself is delivered and not how additional supporting documentation may be accessed, e.g. a F-F teaching programme may use an online platform to upload course syllabus but if the teaching itself is deliver in person this this would classified as F-F. |
| **13. Description** | Brief description of the CBI. |
| **14. Content and curriculum** | High-level summary of the information provided on the content and structure of the CBI, e.g. description in text of CBI, table of curriculum components, online supplementary file on core competencies. |
| **15. Duration** | The time commitment required to undertake the CBI (e.g. ‘one day’, ‘two weeks’). Please denote as ‘NR’ if not reported or not clear. |
| **16. Entry requirements** | Do the authors explicitly state that there are specific entry requirements to undertake the CBI? Entry requirements are defined here as: *‘selective criteria that individuals must meet in order to be accepted on the CBI’* - this could include one or more of the following:   - level of educational attainment and/or grades (e.g. only available to postdoctoral level individuals); - professional background or discipline, e.g. a CBI may only be available to specific professions; - membership of, or affiliation with, a specific organisation or group; - attendance at a specific conference or meeting; - restrictions imposed in relation to funding, e.g. an individual may not be able to apply for a CBI if they have already received D&I-related funding; - the completion of an application form and/or supporting statement which then goes through a competitive review process; - an interview to assess individual suitability for the CBI; - any other information relating to eligibility criteria to undertake the CBI. |
| **17. Number** | Number of students that have completed and/or enrolled in the CBI (if reported) at the time of publication. |
| **18. Evaluation** | Brief summary of key evaluative information (if any) relating to the CBI. Evaluation information can be quantitative and/or qualitative and must be in the form of delegates’ or faculty’ evaluations of the CBI (e.g. how interesting or useful it was). Summary statements of students’ or faculty’ evaluations can also be included, e.g. ‘*overall findings showed the students rated the course positively’*. |
| **19. Cost** | If the CBI is free, denote this as ‘free’. If is not reported or not clear, denote as ‘NR’. |
| **20. Additional comments** | Additional comments relating to the CBI that have not been covered in criteria 5-19 you feel are pertinent to raise. |
